# Public Health Palliative Care and Public Palliative Care Education

**DOI:** 10.3390/healthcare11050745

**Published:** 2023-03-03

**Authors:** Georg Bollig, John P. Rosenberg

**Affiliations:** 1 Department of Anesthesiology, Intensive Care, Palliative Medicine and Pain Therapy, HELIOS Klinikum, 24837 Schleswig, Germany; 2 Last Aid Research Group International (LARGI), 24837 Schleswig, Germany; 3 School of Health, University of the Sunshine Coast, Caboolture, QLD 4510, Australia

This Special Issue, “Public Health Palliative Care (PHPC) and Public Palliative Care Education (PPCE)”, highlights recent advances and challenges in PHPC and PPCE. The articles demonstrate the breadth and diversity of local responses from across the globe, including Europe, Asia, Australia and South America. The COVID-19 pandemic clearly shows how collaboration between healthcare services and communities is an essential component of public health. This collaboration, initially described in the *Ottawa Charter for Health Promotion* [1] and the growing body of literature about public health approaches to palliative and end-of-life care, demonstrates the importance of the broad implementation of palliative care in all communities and societies.

If most people want to die at home, the public must take a role in the support of seriously ill and dying people. The participation of the public is essential to enable more people to die at home. According to Allan Kellehear’s “95% rule” [2], seriously ill and dying people spend little more than 5% of their final year of life in the direct care of healthcare services; the other 95% relies upon relatives, friends or others without healthcare backgrounds to provide support. Public health palliative care (PHPC) and public palliative care education (PPCE) approaches attempt to address this provision of support.

However, public awareness of palliative care is generally low, and although public education has been recommended by a number of authors, PPCE is rarely implemented in most communities [3,4,5,6,7]. Often, this education—like clinical care—is provided by healthcare services with familiarity with the issues of dying, death and loss. Although this is not unreasonable, it is more consistent with the principles and practices of PHPC to provide such education in partnerships between services and communities, demonstrating responses to local needs.

Five papers highlight different aspects and experiences of the implementation of the Last Aid Course (LAC) from different countries and settings. LACs demonstrate a standardised approach to PPCE that has been implemented in 20 countries worldwide, including Germany, Switzerland, Scotland, Lithuania, Australia, Canada and Brazil. LAC was introduced in 2014 to educate the public about death, dying and end-of-life care, to encourage and to empower people to participate in end-of-life care at home, and to enhance the public discussion about these themes [3,8].

Mueller and colleagues report on LACs in a German university hospital [9], where they were very well accepted by the whole staff (both healthcare and non-healthcare professions) in general, but healthcare staff sought extended content and opportunity for group discussions. These findings led to the formation of an extended “LAC Professional” for healthcare professionals comprising ten teaching hours [10]. In Slovenia, Zelko et al. demonstrate that web-based delivery is very well received and favourably rated by LAC participants [11]. They recommend that the LAC should also address the cultural diversity and characteristics of local settings and populations; they describe early experiences with an LAC for deaf people. Macaden at al. report from Scotland that LAC could serve as the foundational core training to promote death literacy in communities to enhance personal knowledge, skills and confidence of community members [12].

In a cross-border pilot study from Denmark and Germany, Bollig et al. showed that LAC participants find individual differences more important than cultural differences in end-of-life care [13]. They describe differences in regulations and organisation of services across the border that people need to be informed about; some suggest the topics of organisation and support across the border, religions and cultures, and supporting people in grief could be added to the LAC. This pilot study has influenced the work of the revision of LAC content during the 3rd International Last Aid Conference in Maribor, 2022. Interviews with German participants of the LAC presented by Giehl et al. inform us about the effects that LAC can have on palliative care provision at home and empowerment of participants [14]. The LAC helps to provide knowledge, guidance and recommendations for action in palliative care situations.

These articles on LACs add to our understanding of the impact of LAC training of both the public and professionals. They show that the LAC concept is feasible in different settings in a range of countries. Indeed, LAC may contribute to increased death literacy in communities, where the development of personal skills to strengthen community action underpins PHPC [15]. In the context of Compassionate Communities, LACs can be seen as practical support offered by fellow citizens to relieve suffering.

Recognition of palliative care as everyone’s responsibility also enables universities to play a role in the development of Compassionate Communities. Librada Flores et al. recognise that, with minimal death awareness in universities, a university that sensitises, trains and mobilises students and professionals to develop care networks around people at the end of life is highly beneficial [16]. Including universities as partners in Compassionate Communities may contribute to social change with the sensitisation of future leaders educated there.

Aoun and her colleagues report on a large community-focused study of bereaved and current carers, and patients, of palliative care [17]. They observe that those with the greatest awareness of palliative care are the ‘winners’ in receiving best quality care and the ‘losers’ were the opposite; however, in both cases, wider social networks are key to providing appropriate support. The importance of communities supporting their members in times of need is very clear, and this reiterates the necessity of public awareness. This is perhaps most clear in communities with a strong, shared cultural identity. Kroik et al. explore this in the indigenous Sámi of Fennoscandia [18]. Where community coherence is high, capacity for well-functioning palliative and end-of-life care is increased; yet the greatest challenge lies in the establishment of supportive interactions with healthcare services. Developing meaningful care relies upon this partnership.

The implementation of the Compassionate Cities charter [19] in downtown Taipei, Taiwan, guided the development of a compassionate community in a local district [20]. A culturally sensitive and holistic program was developed using health and social services, universities and community representatives, which enabled a sustainable approach to community connection and greatly increased awareness of palliative and end-of-life care issues. In South Korea, Bae and Kim also demonstrate the importance of regional knowledge in designing appropriate responses to local needs [21].

Interestingly, this clear sense of common purpose can be created between previously disparate groups. Kleijberg and his colleagues explored the application of the arts to intergenerational groups where community partners, children and older adults were drawn together for intentional ‘play’ [22]. These activities engaged with end-of-life issues, and illustrate a powerful alternative to didactic pedagogy in building knowledge and connections.

Rumbold and Aoun’s review of the literature provides an informative overview of the consumer experience of palliative care, including consumer contribution to service and policy design [23]. Arguably, it is from a community where citizens’ engagement with palliative and end-of-life care issues is high that these experiences are more positive; however, this review demonstrates minimal use of consumer perspectives in the models of palliative care. This is true not only in the low usage of generalised evidence but also in the notable absence of community representation in the co-creation of services.

This Special Issue adds to the growing body of knowledge, based on practice, theory and research, about PHPC and PPCE. It is clear that global perspectives of PHPC are best expressed through both international, national and local activities including PPCE, such as Last Aid. Partnerships between healthcare services, civic organisations and communities are essential to increase awareness, skills and practice. It is evident that PHPC and PPCE are needed to improve palliative care support by the public in communities. These examples show that encouraging work on this important theme is ongoing worldwide, although more effort for implementation and research is needed.
